# Calcium Signaling Is Involved in Cadmium-Induced Neuronal Apoptosis via Induction of Reactive Oxygen Species and Activation of MAPK/mTOR Network

**DOI:** 10.1371/journal.pone.0019052

**Published:** 2011-04-22

**Authors:** Baoshan Xu, Sujuan Chen, Yan Luo, Zi Chen, Lei Liu, Hongyu Zhou, Wenxing Chen, Tao Shen, Xiuzhen Han, Long Chen, Shile Huang

**Affiliations:** 1 Department of Biochemistry and Molecular Biology, Feist-Weiller Cancer Center, Louisiana State University Health Sciences Center, Shreveport, Louisiana, United States of America; 2 Jiangsu Province Key Laboratory for Molecular and Medical Biotechnology, College of Life Sciences, Nanjing Normal University, Nanjing, People's Republic of China; Enzo Life Biosciences, United States of America

## Abstract

Cadmium (Cd), a toxic environmental contaminant, induces oxidative stress, leading to neurodegenerative disorders. Recently we have demonstrated that Cd induces neuronal apoptosis in part by activation of the mitogen-activated protein kineses (MAPK) and mammalian target of rapamycin (mTOR) pathways. However, the underlying mechanism remains elusive. Here we show that Cd elevated intracellular calcium ion ([Ca^2+^]_i_) level in PC12, SH-SY5Y cells and primary murine neurons. BAPTA/AM, an intracellular Ca^2+^ chelator, abolished Cd-induced [Ca^2+^]_i_ elevation, and blocked Cd activation of MAKPs including extracellular signal-regulated kinase 1/2 (Erk1/2), c-Jun N-terminal kinase (JNK) and p38, and mTOR-mediated signaling pathways, as well as cell death. Pretreatment with the extracellular Ca^2+^ chelator EGTA also prevented Cd-induced [Ca^2+^]_i_ elevation, MAPK/mTOR activation, as well as cell death, suggesting that Cd-induced extracellular Ca^2+^ influx plays a critical role in contributing to neuronal apoptosis. In addition, calmodulin (CaM) antagonist trifluoperazine (TFP) or silencing CaM attenuated the effects of Cd on MAPK/mTOR activation and cell death. Furthermore, Cd-induced [Ca^2+^]_i_ elevation or CaM activation resulted in induction of reactive oxygen species (ROS). Pretreatment with BAPTA/AM, EGTA or TFP attenuated Cd-induced ROS and cleavage of caspase-3 in the neuronal cells. Our findings indicate that Cd elevates [Ca^2+^]_i_, which induces ROS and activates MAPK and mTOR pathways, leading to neuronal apoptosis. The results suggest that regulation of Cd-disrupted [Ca^2+^]_i_ homeostasis may be a new strategy for prevention of Cd-induced neurodegenerative diseases.

## Introduction

Cadmium (Cd), a toxic transition metal, which can be released from cigarette smoking, smelting and refining of metals, and burning of chemical fuels and municipal wastes, results in pollution of air, water, and soil [1]. As the half-life of Cd in human body is about 15–20 years [1], chronic exposure to a Cd-contaminated environment or food chain may cause accumulation of Cd in various human organs, such as kidney [2], liver [2], [3], lung [4], [5], testis, bone and brain [6], [7], thereby leading to their damage. Clinical data have shown that Cd contributes to neurological disorders such as learning disabilities and hyperactivity in children [8], [9], olfactory dysfunction and neurobehavioral defects in attention, psychomotor speed, and memory in workers exposed to Cd [7], [10]. Increasing evidence has demonstrated that Cd is a possible etiological factor of neurodegenerative diseases, such as Parkinson's disease, Alzheimer's disease and amyotrophic lateral sclerosis [11]–[13].

Calcium is a ubiquitous intracellular signal responsible for controlling numerous cellular processes including cell proliferation, differentiation, and survival/death [14]. Studies have shown that Cd disrupts intracellular free calcium ([Ca^2+^]_i_) homeostasis, leading to apoptosis in a variety of cells, such as skin epidermal cells [15], hepatic cells [16], [17], lymphoblastoid cells [16], mesangial cells [18]–[20], renal tubular cells [21], [22], astrocytes [23], NIH 3T3 cells [24], thyroid cancer cells [25], and thymocytes [26]. As a second messenger, Ca^2+^ mediates physiological responses of neurons to neurotransmitters and neurotrophic factors [27]–[29]. It has been described that elevation in cytoplasmic Ca^2+^ levels activates the mitogen-activated protein kinase (MAPK) cascade [15], [19] and the phosphatidylinositol 3′-kinase (PI3K)-Akt pathway [29]. Ca^2+^ is also critical for amino acid-mediated activation of mammalian target of rapamycin (mTOR) [30]. Activation of MAPK and/or mTOR pathways may promote cell survival or cell death, depending on stimuli [31]–[33]. Recently, we have demonstrated that Cd-induced neuronal apoptosis is partially associated with activation of the signaling pathways involving c-Jun N-terminal kinase (JNK) and extracellular signal-regulated kinase 1/2 (Erk1/2), as well as Akt/mTOR in neuronal (PC12 and SH-SY5Y) cells [34]–[36]. However, little is known about the role of Ca^2+^ signaling in Cd-mediated activation of MAPK/mTOR pathways and apoptosis in neuronal cells.

Increasing evidence indicates that Cd-induced neuronal toxicity is due to induction of reactive oxygen species (ROS), leading to oxidative stress [23], [35]–[37]. Under pathological conditions, excessive amounts of ROS induced by Cd can modify proteins, lipids and DNA, alter their functions, and activate related signaling pathways [10], [35], [38]–[42]. For example, Cd activates the MAPK pathway by induction of ROS generation, which not only activates the upstream kinases of Erk1/2 and JNK, but also inhibits negative regulators, protein phosphatase 2A (PP2A) and protein phosphatase 5 (PP5), leading to apoptosis of neuronal cells [35]. The data suggest that ROS-induced apoptosis is likely to be a central mechanism of Cd-induced neuronal cell death. It has been described that Cd-induced ROS is related to [Ca^2+^]_i_ elevation in various types of non-neuronal cells [16], [26], [43], [44]. This prompted us to study whether Cd induces oxidative stress by disrupting [Ca^2+^]_i_ homeostasis in neuronal cells.

Here we show that Cd-induced neuronal apoptosis is associated with its induction of [Ca^2+^]_i_ elevation in PC12, SH-SY5Y cells and primary murine neurons. Consequently, Cd-elevated [Ca^2+^]_i_ induces ROS, and activates MAPK and mTOR pathways, leading to neuronal cell death.

## Materials and Methods

### Chemicals

Cadmium chloride (Sigma, St. Louis, MO, USA) was dissolved in sterile distilled water to prepare the stock solutions (0–20 mM), aliquoted, and stored at room temperature. Fluo-3/AM and Fluo-4/AM were purchased from Invitrogen (Grand Island, NY, USA). Poly-D-lysine (PDL), and ethylene glycol tetra-acetic acid (EGTA) were from Sigma. 1,2-bis-(o-Aminophenoxy)-ethane- N,N,N′,N′-tetraacetic acid, tetraacetoxymethyl ester (BAPTA/AM) was purchased from Enzo Life Sciences International (Plymouth Meeting, PA, USA). Trifluoperazine dihydrochloride (TFP) and 5-(and-6)-chloromethyl-2′,7′-dichlorodihydrofluorescein diacetate (CM-H_2_DCFDA) were from MP Biomedicals (Solon, OH, USA).

### Cell culture

Rat pheochromocytoma (PC12) and human neuroblastoma (SH-SY5Y) cell lines were purchased from American Type Culture Collection (Manassas, VA, USA), and were used for no more than 10 and 20 passages, respectively. PC12 cells were grown in antibiotic-free Dulbecco's modified Eagle medium (DMEM) (Mediatech, Herndon, VA, USA) supplemented with 10% horse serum and 5% fetal bovine serum (FBS) (Hyclone, Logan, UT, USA), whereas SH-SY5Y cells were grown in antibiotic-free DMEM supplemented with 10% FBS. Cells were trypsinized with 0.05% Trypsin-EDTA (Invitrogen, Grand Island, NY, USA), sub-cultured, and maintained in a humid incubator (37°C, 5% CO_2_).

Primary murine neurons were isolated from mice as described [45]. Isolated cells were seeded at a density of 2×10^6^ cells/well in a 6-well plate coated with 10 µg/ml PDL in NEUROBASAL™ Media (Invitrogen) supplemented with 2% B27 Supplement (Invitrogen), 2 mM glutamine (Invitrogen), 1 mM sodium pyruvate (Invitrogen), 5 µg/ml insulin (Sigma), and 40 µg/ml of gentamicin (Invitrogen), and grown in a humid incubator (37°C, 5% CO_2_). Fresh medium was replaced every 3 days. The cells were used for experiments after 6 days of culture.

### Lentiviral shRNA cloning, production, and infection

To generate lentiviral short hairpin RNA (shRNA) to calmodulin (CaM), oligonucleotides containing the target sequences were synthesized, annealed and inserted into FSIPPW lentiviral vector via the EcoR1/BamH1 restriction enzyme site, as described previously [46]. Oligonucleiotides used were: sense: 5′-AATTCCCGGATGGAGATGGCACTATCTG CAAGAGAGATAGTGCCATCTCCATCCTTTTTG-3′, anti-sense: 5′-GATCCAAA AAGGATGGAGATGGCACTATCTCTCT TGCAGATAGTGCCATCTCCATCCGG G-3′, which were synthesized by Invitrogen. Lentiviral shRNA construct targeting green fluorescnec protein (GFP) was described [46]. To produce lentiviral shRNAs, above constructs were co-transfected together with pMD2.G and psPAX2 (Addgene, Cambridge, MA, USA) to 293TD cells using Lipfectamine™ 2000 reagent (Invitrogen). Each virus-containing medium was collected 36 and 60 h post-transfection, respectively. For use, monolayer cells, when grown to about 70% confluence, were infected with above lentivirus-containing medium in the presence of 8 µg/ml polybrene for 12 h twice at an interval of 6 h. Uninfected cells were eliminated by exposure to 2 µg/ml puromycin for 48 h before use.

### [Ca^2+^]_i_ detection

Cells were seeded at a density of 5×10^5^ cells/well in completed growth medium in a 6-well plate, precoated with (for PC12) or without (for SH-SY5Y) PDL (0.2 µg/ml). Next day, cells were treated with 0–20 µM Cd for 24 h, with 10 and 20 µM Cd for different time (0–24 h), or with/without 10 and 20 µM Cd for 24 h following pre-incubation with/without BAPTA/AM (30 µM) and EGTA (100 µM) for 30 min with 6 replicates of each treatment in PC12 and/or SH-SY5Y cells. The cells were then trypsinized, washed 3 times with PBS, and resuspended in PBS. Subsequently, cell suspensions (100 µl) for [Ca^2+^]_i_ analysis were loaded with 5 µM Fluo-3/AM for 30 min at 37°C in the dark, washed once with PBS to remove the extracellular Fluo-3/AM. PBS, replacing Fluo-3/AM, served as a negative control. Finally, the cells for each example were resuspended in 1 ml PBS, followed by adding the suspension into a 96-well plate (150 µl/well). Fluorescent intensity was recorded by excitation at 488 nm and emission at 535 nm using a Synergy™ 2 Multi-Function Microplate Reader (Bio-Tek Instruments, Winooski, Vermont, USA).

To visualize the effect of Cd on [Ca^2+^]_i_ in neuronal cells, SH-SY5Y cells were seeded at a density of 5×10^5^ cells/well in 6-well plates containing a glass coverslip per well. Next day, cells were treated with 0–20 µM Cd for 24 h, with 10 µM Cd for different time (0–24 h), or with/without 10 µM Cd for 2 and 24 h in the presence or absence of BAPTA/AM (12.5 µM) or EGTA (100 µM), followed by removing medium, and washing cells 3 times with PBS. The cells for [Ca^2+^]_i_ analysis were loaded with 2.5 µM Fluo-4/AM for 60 min at 37°C in the dark, and then washed once with PBS to remove the extracellular Fluo-4/AM. Finally, calcium imaging was acquired with a Nikon Eclipse TE2000-U inverted fluorescence microscope (Melville, NY, USA) equipped with a digital camera.

### Cell viability assay and morphology

Cells were seeded at a density of 1×10^4^ cells/well in a flat-bottomed 96-well plate, precoated with (for PC12 cells and primary neurons) or without (for SH-SY5Y) PDL. Next day, cells were treated with 0–20 µM Cd for 24 h, with 10 and 20 µM Cd for different time (0–24 h), or with/without 10 and 20 µM Cd for 24 h following pre-incubation with/without BAPTA/AM (30 µM for PC12, 12.5 µM for SH-SY5Y, and 20 µM for primary neurons), 100 µM EGTA, or TFP (50 µM for PC12, 10 µM for SH-SY5Y, and 20 µM for primary neurons) for 30 min with 4–6 replicates of each treatment. Subsequently, each well was added 20 µl of one solution reagent using CellTiter 96 AQ_ueous_ One solution Cell Proliferation Assay kit (Promega, Madison, WI, USA), and incubated for 3 h. Cell viability was determined by measuring the optical density (OD) at 490 nm using a Wallac 1420 Multilabel Counter (Perkin-Elmer Life Sciences, Wellesley, MA, USA).

For cell morphological analysis, cells were seeded at a density of 5×10^5^ cells/well in a 6-well plate as described above. Next day, cells were exposed to Cd (10 and 20 µM) in the presence or absence of BAPTA/AM, EGTA, or TFP at indicated concentrations. After incubation for 24 h, images were taken with a Nikon Eclipse TE2000-U inverted phase-contrast microscope (Melville, NY, USA) (200×) equipped with a digital camera.

### ROS detection

The ROS level was measured using CM-H_2_DCFDA, as described [35]. Briefly, primary neurons and SH-SY5Y cells were seeded at a density of 1×10^4^ cells/well in a 96-well plate, respectively. The next day, cells were treated with Cd (10 and 20 µM) for 24 h in the presence or absence of BAPTA-AM, EGTA, or TFP at indicated concentrations, followed by incubation with CM-H_2_DCFDA for 3 h. Fluorescent intensity was recorded by excitation at 485 nm and emission at 535 nm using a Wallac 1420 Multi-label counter (Perkin-Elmer Life Sciences, Wellesley, MA).

### Western blot analysis

Western blot analysis was performed as described [34]. The following antibodies were used: phospho-Erk1/2 (Thr202/Tyr204), phospho-p38 (Thr180/Tyr182), phospho-Akt (Ser473), phospho-S6K1 (Thr389), phospho-mTOR (Ser2448), mTOR, 4E-BP1, caspase-3, cleaved caspase-3 (Asp175), cleaved PARP (Asp214) (Cell Signaling Technology, Beverly, MA, USA), JNK1, phospho-JNK (Thr183/Tyr185), c-Jun, phospho-c-Jun (Ser63), Erk2, p38, Akt, S6K1, CaM (Santa Cruz Biotechnology, Santa Cruz, CA, USA), β-tubulin (Sigma, MO), goat anti-rabbit IgG-horseradish peroxidase (HRP), goat anti-mouse IgG-HRP, and rabbit anti-goat IgG-HRP (Pierce, Rockford, IL, USA). Enhanced chemiluminescence solution was from Pierce.

### Statistical analysis

Results were expressed as mean values ± standard error (mean ± S.E.). Statistical analysis was performed by Student's *t* test (STATISTICA, Statsoft Inc., Tulsa, OK, USA). A level of *P*<0.05 was considered to be statistically significant.

## Results

### Cd induces intracellular [Ca^2+^]_i_ elevation in neuronal cells

**Figure 1:**
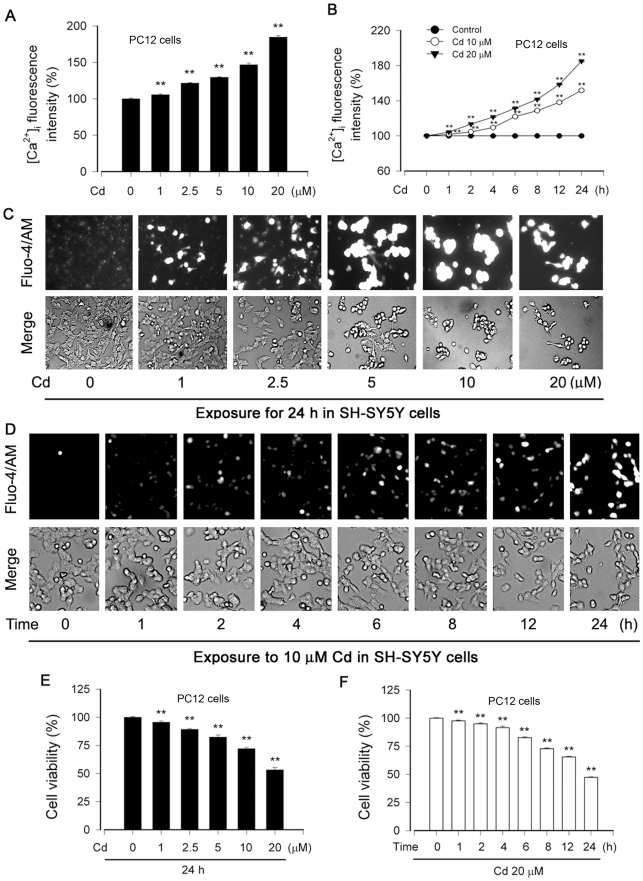
Cd-induced neuronal apoptosis is associated with induction of [Ca^2**+**^]_i_ elevation. (A and B) PC12 cells were treated with 0–20 µM Cd for 24 h, or with 0, 10 and 20 µM Cd for 0–24 h, and then loaded with 5 µM Fluo-3/AM for 30 min at 37°C in the dark, followed by measurement of [Ca^2+^]_i_ fluorescence intensity, as described in Materials and Methods. (C and D) SH-SY5Y cells were exposed to 0–20 µM Cd for 24 h, or 10 µM Cd for 0–24 h, and then loaded with 2.5 µM Fluo-4/AM for 60 min at 37°C in the dark, followed by recording of the images under a fluorescence microscope. (E and F) Cell viability of PC12 cells, treated with 0–20 µM Cd for 24 h or with 20 µM Cd for 0–24 h, was evaluated by one solution assay. Results are presented as mean ± SE; *n* = 6. ^**^ *P*<0.01 difference vs. control group.

To determine the role of calcium signaling in Cd-induced neuronal apoptosis, PC12 and SH-SY5Y cells, respectively, were treated with 0–20 µM Cd for 24 h, or with 10 and 20 µM Cd for 0–24 h. Subsequently, [Ca^2+^]_i_ was measured with a calcium indicator dye, Fluo-3/AM or Fluo-4/AM. We found that treatment with Cd (0–20 µM) resulted in a concentration-dependent increase of [Ca^2+^]_i_ in PC12 cells (Fig. 1A). Cd also induced a time-dependent elevation of [Ca^2+^]_i_ in the cells during the period of 24 h (Fig. 1B). Similarly, Cd markedly elicited high [Ca^2+^]_i_ fluorescence intensity in a concentration- and time-dependent manner in SH-SY5Y cells by fluorescence microscopy (Fig. 1C and D). Furthermore, Cd-elevated [Ca^2+^]_i_ level was consistent with decreased cell viability (Fig. 1E and F) or increased apoptosis of PC12 and SH-SY5Y cells [35], suggesting that Cd-induced neuronal apoptosis might be associated with its induction of [Ca^2+^]_i_ elevation.

### Cd elevated [Ca^2+^]_i_ activates MAPK and mTOR pathways leading to apoptosis in neuronal cells

**Figure 2:**
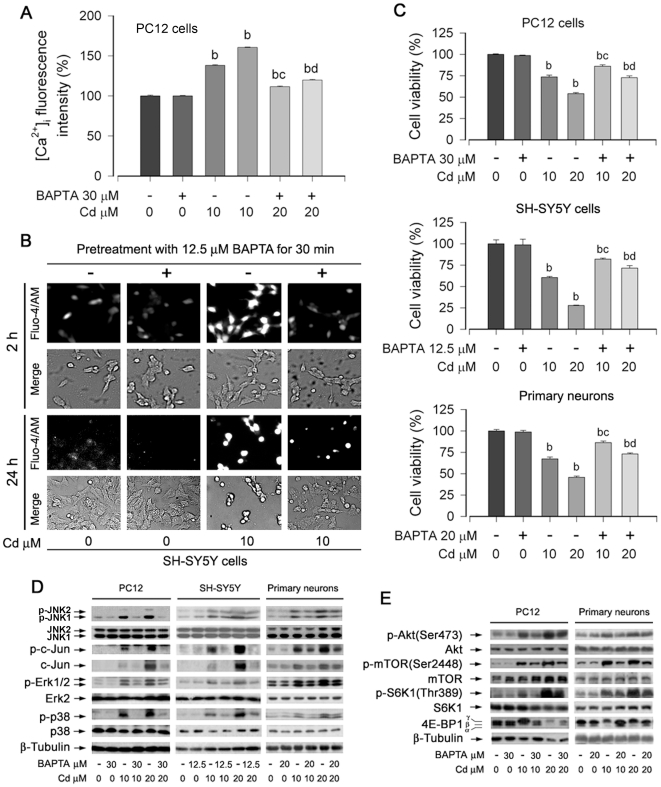
Cd activates MAPK/mTOR pathways and neuronal apoptosis via induction of [Ca^2**+**^]_i_ elevation. Indicated cells were pretreated with/without BAPTA/AM at indicated concentrations for 30 min, and then exposed to Cd (10 and/or 20 µM) for 24 h (A–C) or 4 h (D, E). (A) [Ca^2+^]_i_ in PC12 cells was determined by measuring Fluo-3/AM-labeled fluorescent intensity, as described in Materials and Methods. (B) [Ca^2+^]_i_ was stained with Fluo-4/AM and visualized by fluorescence microscopy in SH-SY5Y cells. (C) Cell viability for PC12, SH-SY5Y, and primary neurons was evaluated using one solution assay. (D and E) Indicated cell lysates were subjected to Western blotting using indicated antibodies. The blots were probed for β-tubulin as a loading control. Similar results were observed in at least three independent experiments. Results (A and C) are presented as mean ± SE; *n* = 6. ^b^ *P*<0.01, difference vs. control group; ^c^ *P*<0.01, difference vs. 10 µM Cd group; ^d^ *P*<0.01, difference vs. 20 µM Cd group.

To validate the pivotal role of [Ca^2+^]_i_ elevation in Cd-induced neuronal apoptosis, PC12 cells were pretreated with/without 30 µM BAPTA/AM, an intracellular Ca^2+^ chelator, for 30 min, and then exposed to Cd (10 and 20 µM) for 24 h. We found that pretreatment with BAPTA/AM significantly blocked Cd-triggered [Ca^2+^]_i_ elevation (Fig. 2A). Similar results were also seen in SH-SY5Y cells (Fig. 2B). One-solution assay showed that BAPTA/AM partially prevented Cd-decreased cell viability in PC12, SH-SY5Y cells and primary neurons (Fig. 2C). The results demonstrate that Cd induces neuronal apoptosis through induction of [Ca^2+^]_i_ elevation.

Recently we have demonstrated that Cd induces apoptosis of PC12 and SH-SY5Y cells via activation of MAPK and mTOR signaling network [34]. To examine whether Cd-induced [Ca^2+^]_i_ elevation is correlated to the activation of MAPK and mTOR pathways, PC12, SH-SY5Y, and primary neurons were preincubated with/without BAPTA/AM for 30 min, followed by treatment with Cd (10 and 20 µM) for 4 h. Western blot analysis showed that BAPTA/AM significantly blocked Cd-induced phosphorylation of JNK, Erk1/2, and p38 MAPK (Fig. 2D), as well as phosphorylation of mTOR, and mTOR-mediated S6K1 and 4E-BP1 (Fig. 2E). It should be mentioned that phosphorylation state of 4E-BP1 was detected with an antibody to 4E-BP1. Phosphorylation of 4E-BP1 decreases its electrophoretic mobility during sodium dodecyl sulfate-polyacrylamide gel electrophoresis [34]. As shown in Fig. 2E, Cd increased phosphorylation of 4E-BP1, as indicated by the increase in the intensity of the uppermost band γ and by the decrease in the higher mobility band α and β that corresponds to a less phosphorylated form of 4E-BP1. Consistently, we also noticed that Cd-activated Akt as the main upstream mediator of mTOR signaling was also partially abrogated by BAPTA/AM in PC12 cells and primary neurons (Fig. 2E). These results unveil that Cd induction of [Ca^2+^]_i_ elevation activates the MAPK and mTOR pathways, triggering apoptosis of the neuronal cells.

### Cd-induced extracellular Ca^2+^ influx elevates [Ca^2+^]_i_ contributing to neuronal apoptosis via activation of MAPK and mTOR pathways

**Figure 3:**
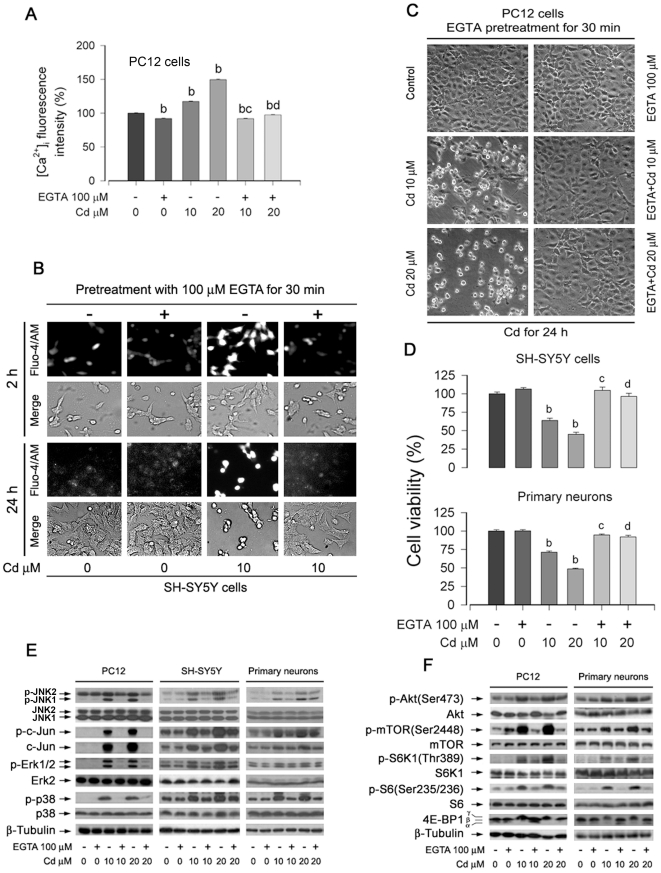
Cd-induced extracellular Ca^2**+**^ influx elevates [Ca^2**+**^]_i_ contributing to neuronal apoptosis via activation of MAPK and mTOR pathways. Indicated cells were pretreated with or without 100 µM EGTA for 30 min, and then exposed to Cd (10 and/or 20 µM) for 24 h (A–D) or 4 h (E, F). (A and B) [Ca^2+^]_i_ fluorescent intensities were evaluated as described in Materials and Methods. (C) Morphology of PC12 cells was assessed using a Nikon Eclipse TE2000-U inverted phase-contrast microscope (200×) equipped with digital camera. (D) Cell viability in SH-SY5Y cells and primary neurons was evaluated by one solution assay. (E and F) Indicated cell lysates were subjected to Western blotting using indicated antibodies. The blots were probed for β-tubulin as a loading control. Similar results were observed in at least three independent experiments. Results (A, D) are presented as mean ± SE; *n* = 6. ^b^ *P*<0.01, difference vs. control group; ^c^ *P*<0.01, difference vs. 10 µM Cd group; ^d^ *P*<0.01, difference vs. 20 µM Cd group.

To investigate the role of extracellular Ca^2+^ in Cd-induced neuronal apoptosis, EGTA, an extracellular Ca^2+^ chelator, was utilized. As shown in Fig. 3A and B, pretreatment with 100 µM EGTA for 30 min almost completely abolished [Ca^2+^]_i_ elevation induced by 10 and 20 µM Cd in PC12 or SH-SY5Y cells. Consistently, we observed that Cd alone (10 and 20 µM) induced cell roundup and shrinkage, and EGTA itself did not alter cell shape. However, EGTA obviously blocked Cd-induced morphological change (Fig. 3C). One-solution assay showed that EGTA significantly attenuated Cd-decreased cell viability in SH-SY5Y cells, and primary neurons (Fig. 3D). The findings suggest that Cd elevates [Ca^2+^]_i_ at least in part by increasing extracellular Ca^2+^ influx, leading to apoptosis of neuronal cells.

To determine the effects of extracellular Ca^2+^ on MAPK and mTOR pathways, PC12, SH-SY5Y, and primary neurons were pretreated with/without EGTA for 30 min, and then exposed to Cd for 4 h, followed by Western blot analysis. We found that EGTA blocked Cd-induced phosphorylation of Erk1/2, JNK, and p38 MAPK (Fig. 3E), as well as phosphorylation of Akt/mTOR pathways (Fig. 3F). The results indicate that Cd may elevate [Ca^2+^]_i_ in neuronal cells partially by increasing extracellular Ca^2+^ influx, leading to neuronal apoptosis via activation of MAPK and mTOR pathways.

### Cd-elevated [Ca^2+^]_i_ activates MAPK/mTOR pathways and apoptosis in neuronal cells through calcium-binding protein CaM

**Figure 4:**
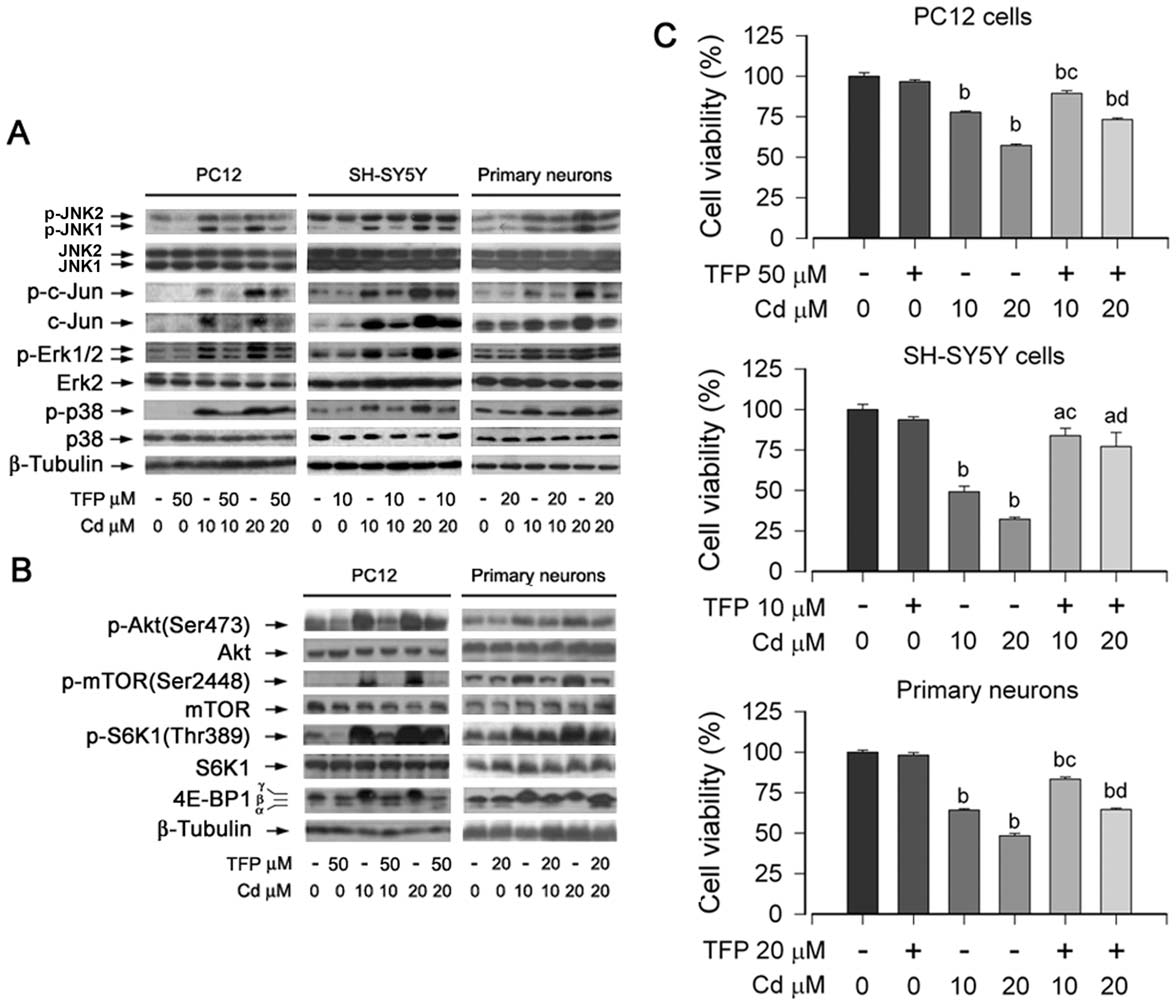
Inhibition of CaM by TFP attenuates Cd activation of MAPK/mTOR and apoptosis in neuronal cells. Indicated cells were pretreated with/without TFP at indicated concentrations for 30 min, and then exposed to Cd (10 and 20 µM) for 4 h (A, B) or 24 h (C). (A and B) Indicated cell lysates were subjected to Western blotting using indicated antibodies. The blots were probed for β-tubulin as a loading control. Similar results were observed in at least three independent experiments. (C) Cell viability for indicated cells was evaluated using one solution assay. Results are presented as mean ± SE; *n* = 6. ^a^ *P*<0.05, ^b^ *P*<0.01, difference vs. control group; ^c^ *P*<0.01, difference vs. 10 µM Cd group; ^d^ *P*<0.01, difference vs. 20 µM Cd group.

CaM, a multifunctional Ca^2+^-binding protein, acts as a transducer of the intracellular calcium signal for a variety of cellular events, including apoptosis [14], [29]. Many proteins that CaM binds cannot bind calcium themselves, and have to use CaM as a calcium sensor and signal transducer [14], [29]. We proposed that Cd-elevated [Ca^2+^]_i_ activates MAPK/mTOR pathways and induces neuronal apoptosis through CaM. To test this hypothesis, PC12 and SH-SY5Y cells were pretreated with CaM antagonist TFP (50 µM for PC12 or 10 µM for SH-SY5Y) for 30 min, and then exposed to Cd (10, 20 µM) for 24 h, followed by Western blotting and cell viability assay. The results showed that TFP partially blocked Cd-induced phosphorylation of Erk1/2, JNK, and p38 in PC12, SH-SY5Y and primary neurons (Fig. 4A). Cd-activated phosphorylation of Akt, mTOR, S6K and 4E-BP1 in PC12 cells and primary neurons was also significantly reduced by TFP (Fig. 4B). One-solution assay revealed that TFP significantly attenuated Cd-decreased cell viability in PC12, SH-SY5Y and primary neurons (Fig. 4C).

**Figure 5:**
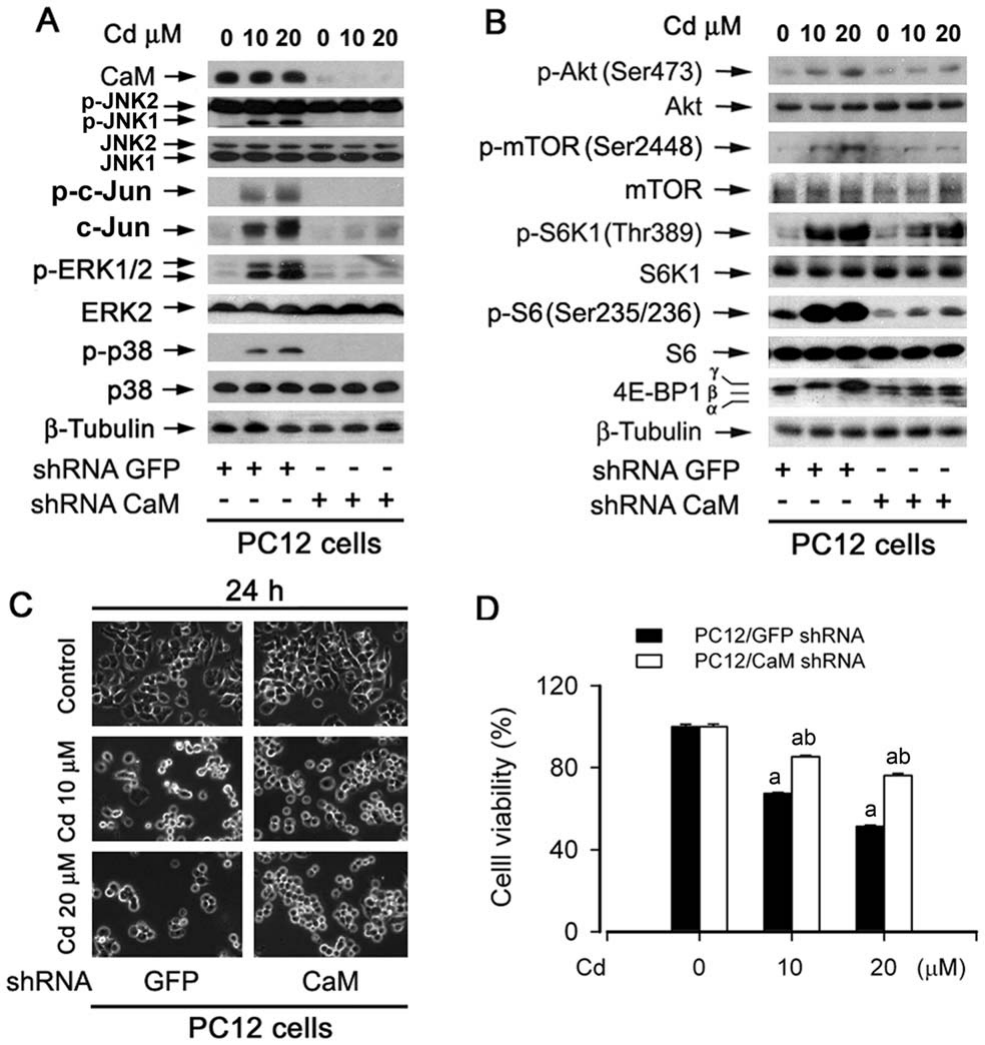
Downregulation of CaM attenuates Cd activation of MAPK/mTOR pathways and apoptosis in neuronal cells. PC12 Cells infected with lentiviral shRNAs to CaM and GFP, respectively, were exposed to Cd (0–20 µM) for 4 h (A,B) or 24 h (C,D), followed by Western blotting with indicated antibodies (A, B), cell morphological analysis (C) or cell viability assay (D), as described in Materials and Methods. Note: CaM was downregulated by ∼90% by lentiviral shRNA to CaM, compared with the control (lentiviral shRNA to GFP), by densitometry using NIH Image J. Results are presented as mean ± SE; *n* = 6. ^a^ *P*<0.01, difference vs. control group; ^b^ *P*<0.01, CaM shRNA group vs. GFP shRNA group.

To substantiate the role of CaM in Cd-induced activation of MAPK/mTOR and apoptosis in neuronal cells, CaM was silenced by RNA interference technology. As shown in Fig. 5A, lentiviral shRNA to CaM downregulated protein expression of CaM by ∼90% in PC12 cells, compared with the control shRNA to GFP. Silencing CaM remarkably inhibited Cd-induced phosphorylation of MAPK and Akt/mTOR pathways in PC12 cells (Fig. 5A and B). Importantly, downregulation of CaM obviously attenuated Cd inhibition of cell viability (Fig. 5C and D). The results indicate that Cd-elevated [Ca^2+^]_i_ activates MAPK/mTOR network and induces apoptosis in neuronal cells through CaM.

### Cd-elevated [Ca^2+^]_i_ induces ROS, triggering apoptosis of neuronal cells

**Figure 6:**
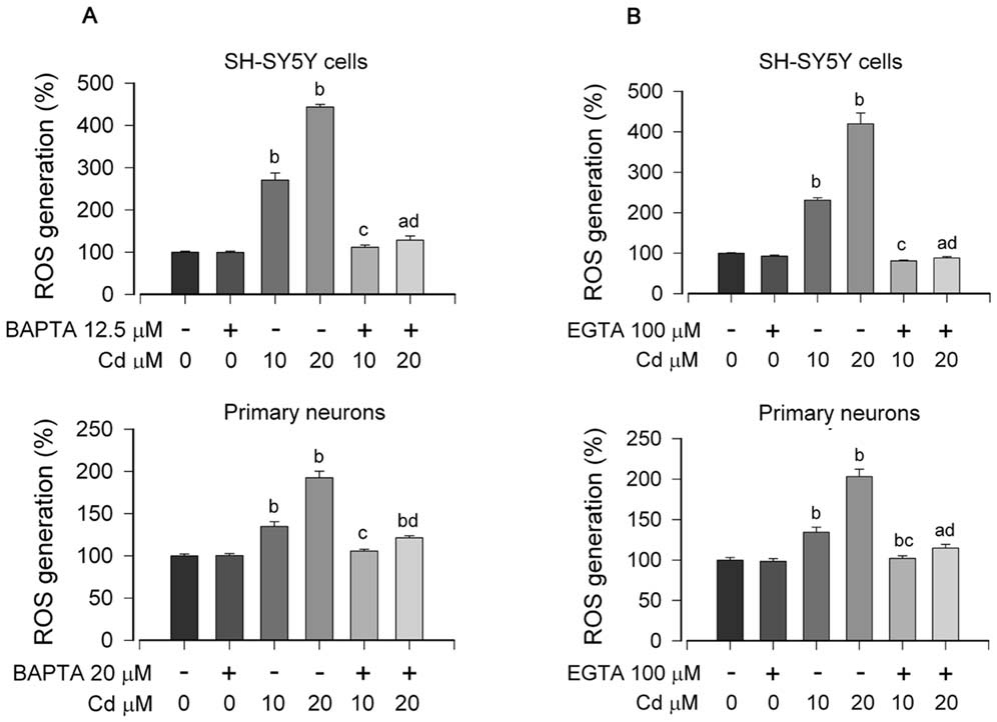
Cd-elevated [Ca^2**+**^]_i_ induces ROS in neuronal cells. Indicated cells were exposed to 0–20 µM Cd for 24 h after pretreatment with/without indicated concentrations of BAPTA/AM (A) or EGTA (B) for 30 min, followed by ROS detection, as described in Materials and Methods. Results are presented as mean ± SE; *n* = 6. ^a^ *P*<0.05, ^b^ *P*<0.01, difference vs. control group; ^c^ *P*<0.01, difference vs. 10 µM Cd group; ^d^ *P*<0.01, difference vs. 20 µM Cd group.

**Figure 7:**
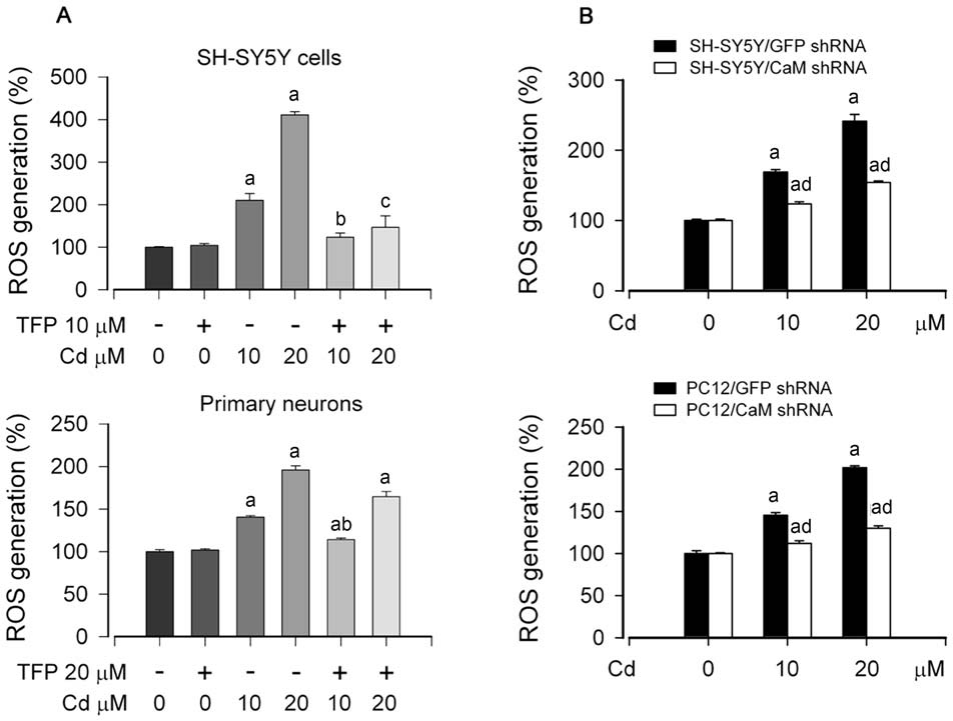
CaM is essential for Cd induction of ROS in neuronal cells. Indicated cells pretreated with/without TFP at indicated concentrations for 30 min (A), or infected with lentiviral shRNAs to CaM and GFP, respectively (B), were exposed to 0–20 µM Cd for 24 h, followed by ROS detection, as described in Materials and Methods. Results are presented as mean ± SE; *n* = 6. ^a^ *P*<0.01, difference vs. control group; ^b^ *P*<0.01, difference vs. 10 µM Cd group; ^c^ *P*<0.01, difference vs. 20 µM Cd group; ^d^ *P*<0.01, CaM shRNA group vs. GFP shRNA group.

**Figure 8:**
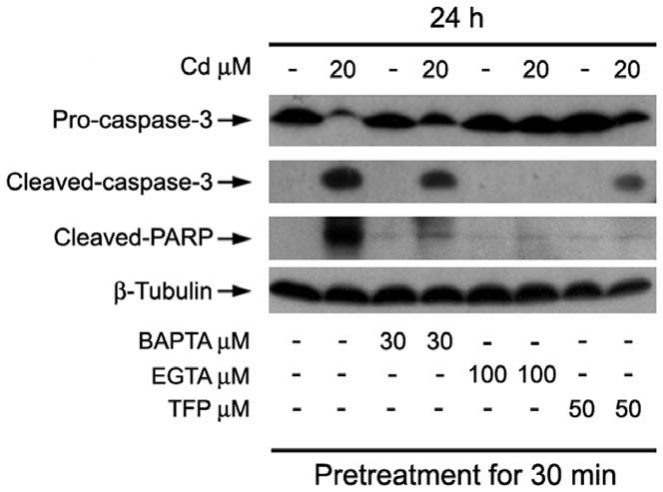
Cd-elevated [Ca^2**+**^]_i_ induces ROS, triggering apoptosis of neuronal cells. PC12 cells, pretreated with/without BAPTA/AM, EGTA, and TFP at indicated concentrations for 30 min, were treated with/without 20 µM Cd for 24 h, followed by Western blot analysis using indicated antibodies. The blots were probed for β-tubulin as a loading control. Similar results were observed in at least three independent experiments.

Recently we have demonstrated that Cd-induced neuronal apoptosis is attributed to induction of ROS [35], [36]. In this study, we have shown that Cd induces apoptosis of PC12 and SH-SY5Y cells due to [Ca^2+^]_i_ elevation (Fig. 1). Therefore, next we sought to test whether Cd-elevated [Ca^2+^]_i_ contributes to ROS induction, leading to cell death. To this end, SH-SY5Y and primary neuron cells were pretreated with/without BAPTA/AM or EGTA for 30 min, respectively, and then exposed to 10 and 20 µM Cd for 24 h. As shown in Fig. 2 and Fig. 3, pretreatment with BAPTA/AM or EGTA inhibited Cd-induced [Ca^2+^]_i_ elevation. Exposure to Cd (10 and 20 µM) for 24 h increased ROS levels by approximately 1.5–4.5 fold in SH-SY5Y cells and by 1.4–2.0 fold in primary neurons, respectively (Fig. 6A and B). Interestingly, pretreatment with BAPTA/AM or EGTA alone did not obviously alter the basal level of ROS, but strikingly attenuated Cd induction of ROS (Fig. 6A and B). Similarly, pretreatment with CaM antagonist TFP also profoundly attenuated Cd induction of ROS in SH-SY5Y and primary neurons (Fig. 7A), suggesting involvement of CaM. This was further confirmed by silencing CaM in PC12 and SH-SY5Y cells (Fig. 7B). In addition, we further noticed that treatment of PC12 cells with 20 µM Cd for 24 h resulted in robust activation of caspase-3, as detected by decreased pro-caspase-3, and increased cleavage of caspase-3 (Fig. 8). Activation of caspase-3 also enhanced cleavage of poly (ADP-ribose) polymerase (PARP) (Fig. 8), indicating apoptosis. As expected, BAPTA/AM, EGTA or TFP obviously attenuated Cd-induced cleavages of caspase-3 and PARP in the cells (Fig. 8), which is agreement with our findings that BAPTA/AM, EGTA or TFP profoundly prevented Cd-induced apoptosis of PC12 cells (Fig. 2, 3 and 4). Similar results were observed in SH-SY5Y cells (data not shown). These data suggest that Cd elevates [Ca^2+^]_i_ level, which induces ROS, triggering apoptosis in neuronal cells.

## Discussion

Calcium has been recognized as a ubiquitous intracellular signal responsible for numerous cellular events, such as growth, proliferation, differentiation, and survival/apoptosis [14]. As a second messenger, Ca^2+^ mediates responses of neurons to neurotransmitters and neurotrophic factors, including cell survival or death signals [27]–[29]. Dysfunction of cellular Ca^2+^ homeostasis induces neuronal cell death, which is implicated in many neurodegenerative disorders, such as Alzheimer's disease and Parkinson's diseases [47]–[50]. Here, for the first time, we present evidence that Cd elevates [Ca^2+^]_i_ level, thereby activating MAPK/mTOR pathways, leading to apoptosis in PC12, SH-SY5Y cells and primary murine neurons. Our results are in line with the previous findings [15]–[26]. It has been described that Cd disrupts [Ca^2+^]_i_ homeostasis, causing apoptosis in a variety of non-neuronal cells, including skin epidermal cells [15], hepatic cells [16], [17], lymphoblastoid cells [16], mesangial cells [18]–[20], renal tubular cells [21], [22], NIH 3T3 cells [24], thyroid cancer cells [25], and thymocytes [26], as well as brain glial cells (astrocytes) [23]. Cd-increased cytoplasmic Ca^2+^ activates p38 in mesangial cells [19] and JNK in skin epidermal cells [15]. Cd-elevated [Ca^2+^]_i_ can also activate PI3K/Akt in thyroid carcinoma cells [25]. In the present study, we found that Cd-elevated [Ca^2+^]_i_ activated JNK, p38, Erk1/2, Akt and mTOR in PC12, SH-SY5Y cells and murine primary neurons. A new question that arises from the current work is whether Cd-elevated [Ca^2+^]_i_ activates MAPKs and PI3K-Akt-mTOR pathways is a cell type context or a general mechanism in all mammalian cells.

Because [Ca^2+^]_i_ increase is usually caused by Ca^2+^ mobilization from intracellular stores and/or Ca^2+^ entry from the extracellular space [14], we investigated the source of [Ca^2+^]_i_ induced by Cd. In this study, we observed that Cd-induced [Ca^2+^]_i_ elevation in PC12 and SH-SY5Y cells was almost completely abolished by EGTA (Fig. 3), an extracellular Ca^2+^ chelator, which renders the inaccessibility of extracellular Ca^2+^ to the cells. Consistently, EGTA also blocked Cd-induced phosphorylation of MAPK and mTOR pathways, and dramatically attenuated the toxicity of Cd in SH-SY5Y and primary neurons (Fig. 3). The findings indicate that Cd may elevate [Ca^2+^]_i_ in neuronal cells in part by increasing extracellular Ca^2+^ influx, leading to neuronal apoptosis via activation of MAPK and mTOR pathways. It is worthy mentioning that during the studies, we also observed that pretreatment with 2-aminoethoxydiphenyl borate, a membrane-permeable inhibitor of inositol trisphosphate receptor [51], markedly attenuated Cd-induced [Ca^2+^]_i_ elevation, and partially blocked Cd-activated Erk1/2, JNK, p38 and mTOR pathways, as well as neuronal apoptosis (data not shown), suggesting that Cd-induced [Ca^2+^]_i_ elevation may involve induction of intracellular release of Ca^2+^ storage as well.

CaM, a Ca^2+^-binding protein, functions as a calcium signal transducer [14]. Many enzymes that cannot bind calcium have to use CaM as a calcium sensor [14], [29]. Inhibition of CaM by the antagonists, such as TFP and Tamoxifen, prevents CD4+ T-cells from HIV-induced apoptosis [52]. However, little is known about the role of CaM in Cd-induced neuronal apoptosis. To better understand how calcium regulates Cd-mediated neurotoxicity, we further studied CaM. Since most recently we have found that Cd upregulates ROS generating enzyme NADPH oxidase 2 and its regulatory proteins [36], originally, we speculated that Cd might activate CaM function not only by increasing Ca^2+^ binding but also by upregulating protein expression of CaM, leading to neuronal apoptosis. However, to our surprise, exposure to Cd (10 or 20 µM) for 24 h did not alter protein expression of CaM in PC12 and SH-SY5Y cells (data not shown). As expected, pretreatment with CaM antagonist TFP, which reduces Ca^2+^ binding to CaM [53], did significantly attenuate Cd-induced activation of MAPK and mTOR pathways, as well as cell death in PC12, SH-SY5Y and primary neurons (Fig. 4). Similarly, silencing CaM with lentiviral shRNA to CaM also remarkably prevented Cd-induced activation of MAPK and mTOR network, as well as cell death in PC12 cells (Fig. 5). Therefore, our data support the notion that Cd induces neuronal apoptosis through Ca^2+^/CaM-mediated activation of MAPK and mTOR pathways.

In the study, we noticed that Cd-induced [Ca^2+^]_i_ elevation did not alter total cellular protein expression of JNK1/2, but preferentially induced p-JNK1 (the lower band), which is particularly obvious in PC12 cells (Fig. 5). Also, activation of JNK1, bot not JNK2 (the upper band), is critical for phosphorylation of c-Jun (Fig. 5). It appears that CaM plays a critical role in this process. This is strongly supported by the findings that silencing CaM by shRNA dramatically blocked Cd-induced p-JNK1, but not p-JNK2, abrogating Cd-induced phosphorylation of c-Jun and attenuating Cd-induced cell death (Fig. 5). The results support the notion that JNK1 and JNK2 are regulated by different mechanisms, and have distinct signaling functions. Similar finding has been documented in myeloid leukemia cells [54]. JNK1 positively regulates vitamin D (1,25-dihydroxyvitamin D_3_)-induced differentiation in HL60 and U937 cells, but JNK2 negatively regulates this process, which is associated with activation of c-Jun and other transcription factors [54]. Furthermore, we also observed that c-Jun cellular protein level is correlated to its phosphorylation status. When c-Jun was phosphorylated, high level of c-Jun was detected (Fig. 2, 3, 4, 5). This is consistent with previous findings that phosphorylation of c-Jun by JNK protects c-Jun from ubiquitination and prolongs its half-life [55].

Currently we do not know what isoforms of p38 MAPK is activated by Cd-induced [Ca^2+^]_i_ elevation. Four isoforms of p38 (α, -β, -γ, and -δ) have been identified [56]. Of importance, various isoforms of p38 have unique cellular functions [56]–[58]. In the study, an antibody to phospho-p38 (Thr180/Tyr182) (Cat.# 9215, Cell Signaling) was used, which cannot differentiate isoforms of p38α, -β, -γ, and -δ. Our previous studies have demonstrated that activation of p38 MAPK is not involved in Cd-induced neuronal cell death [34]. Further studies may not only help identify what isoforms of p38 MAPK is activated by Cd, but also elucidate the potential role of the specific isoforms of p38 activation in neuronal cells.

We are puzzled that Cd activation of Akt/mTOR signaling pathways promotes neuronal cell death. It is commonly accepted that mTOR is a master kinase, which positively regulates protein synthesis, cell growth, proliferation and survival [32], [59]. In our studies, we have found that under different stress conditions, the consequence of activation of Akt/mTOR pathway in neuronal cells is completely different [34]–[36], [45]. In response to hydrogen peroxide, mTOR pathway was persistently (>24 h) inhibited, and overexpression of mTOR attenuated hydrogen peroxide-induced neuronal apoptosis [45], suggesting that certain level of mTOR activation is essential for neuronal cell survival. On the other hand, in response to Cd, mTOR was sustainably (>24 h) activated, and pretreatment with rapamycin, an mTOR inhibitor, blocked Cd-induced phosphorylation of S6K1 and 4E-BP1, and markedly attenuated Cd-induced apoptosis [34]. The results imply that sustained hyper-activation of mTOR is actually not beneficial, but detrimental to neuronal cells, particularly under oxidative stress. As mTOR controls Cap-dependent translation [32], [59], we speculate that Cd activation of mTOR would enhance protein synthesis in the cells, which may consume a lot of energy (ATP) and meanwhile generate much ROS. If mTOR is persistently activated, too much ATP will be consumed and too much ROS will be generated, leading to cell death.

Cd is a well-known inducer of ROS generation in cells [60]. Recently, we have shown that Cd induced ROS generation in a time- and concentration-dependent manner in PC12 and SH-SY5Y cells [35], which causes apoptosis of neuronal cells via activation of MAPKs and mTOR signaling pathways [34]–[36]. However, whether Cd-induced [Ca^2+^]_i_ signaling is involved in these events remains enigmatic. Here, we show that chelation of calcium with BAPTA/AM or EGTA (Fig. 6) or inhibition of CaM with TFP or CaM shRNA (Fig. 7) dramatically attenuated Cd-induced ROS in SH-SY5Y, PC12 or primary neurons. Furthermore, we also observed that BAPTA/AM, EGTA and TFP could obviously reduce cleavages of caspase-3 and PARP in Cd-induced PC12 cells (Fig. 8), which is agreement with our finding that BAPTA/AM, EGTA or TFP was able to strikingly prevent Cd-induced neuronal cell death (Fig. 2 and 3). These data reveal that Cd-induced apoptosis of neuronal cells is triggered by elevated [Ca^2+^]_i_, leading to ROS induction and subsequent activation of caspase signaling pathway.

In summary, here we have shown that Cd-induced [Ca^2+^]_i_ elevation, which was implicated in increased CaM function, induced ROS and activated MAPK and mTOR pathways, thereby leading to caspase-dependent apoptosis of neuronal cells. Cd-induced extracellular Ca^2+^ influx appears to play a critical role in contributing to neuronal apoptosis. Regulation of Cd-disrupted [Ca^2+^]_i_ homeostasis may have a potential for prevention of Cd-induced neurodegenerative diseases.
